# Educational interventions for imposter phenomenon in healthcare: a scoping review

**DOI:** 10.1186/s12909-023-04984-w

**Published:** 2024-01-08

**Authors:** Z Kamran Siddiqui, H. R Church, R. Jayasuriya, T. Boddice, J. Tomlinson

**Affiliations:** 1 Sheffield Teaching Hospitals NHS Foundation Trust, Sheffield, UK; 2 Faculty of Medicine and Health Sciences, University of Nottingham, Nottingham, UK; 3 NHS England Workforce, Training and Education North East and Yorkshire, Sheffield, Yorkshire and Humber UK; 4 Mid Yorkshire Teaching NHS Trust, Wakefield, UK; 5 Faculty of Health, The University of Sheffield, Sheffield, UK; 6 Nottingham University Hospitals NHS Trust, Nottingham, UK

**Keywords:** Imposter phenomenon, Impostor syndrome, Intervention, Workshop, Coaching

## Abstract

**Background:**

Imposter Phenomenon (IP) is a subjective feeling of intellectual fraudulence and self-doubt experienced by individuals in goal-orientated high-achieving professions. The impact of IP within healthcare has been associated with individual physical and mental health and concerns around training, career progression and DEI at an institutional level. To effectively address IP in healthcare, this scoping review aims to explore educational interventions designed to empower high-achieving individuals with the tools needed to confront and overcome IP.

**Methods:**

The scoping review adhered to a predetermined protocol informed by the JBI methodology and PRISMA-ScR guidelines in order to identify educational interventions addressing IP in high-achieving industries. Articles were searched across multiple databases, including MEDLINE (Ovid), PsychINFO, SCOPUS, and Web of Science, alongside grey literature, without imposing any time constraints. A systematic approach including a thematic analysis allowed for a nuanced exploration and interpretation of the identified educational interventions and their impact on addressing IP.

**Results:**

Seventeen articles were incorporated into the review, with the majority originating from the USA and majority being published since 2020. Ten studies targeted healthcare professionals, undergraduate and postgraduate healthcare students. Majority of studies aimed at addressing IP, featured a larger number of female participants than males. Workshops with self-reflection and group-guided exercises to overcome IP were the most popular educational interventions. Coaching and structured supervision were also suggested. Across all papers, three themes emerged for coping strategies: individual, peer-to-peer, and institutional.

**Conclusions:**

This scoping review suggests how group and individual interventions such as workshops, small group discussions and coaching can be used to overcome IP in healthcare. Institutional changes like diversity promotion, supervisor education, and support networks are crucial in addressing IP. Further long term and speciality specific assessments are needed to measure impact. Overall, the review highlights how educational awareness and a variety of strategies can be implemented to create a supportive environment for professionals dealing with IP, promoting their well-being and success.

**Supplementary Information:**

The online version contains supplementary material available at 10.1186/s12909-023-04984-w.

## Background

Imposter Phenomenon (IP), a psychological experience characterised by feelings of intellectual fraudulence despite evident accomplishments, was first described in 1978 by Clance who recognised the phenomena in high-achieving women [1]. IP is experienced as debilitating self-doubt often leading high-achieving individuals to ascribe their success to luck or external factors [2]. High-achieving industries are defined as those that demand exceptional performance, innovation, and competitiveness, fostering a constant pursuit of excellence. IP can be prevalent in such goal-oriented environments, driven by the pressure to meet high standards. Examples of such industries include, but are not limited to, healthcare, technology, business, law, arts, academia, and research. Studies have shown that at least 70% of high-achievers report experiencing the collective symptoms of imposterism: self-doubt, intellectual fraudulence and feelings of fear and failure, at some point during their careers [3, 4]. Furthermore, interpersonal traits, such as maladaptive perfectionism and neuroticism, can contribute to the persistence of IP. Additionally, environmental factors like experiences of discrimination, external negative stereotypes, family dynamics, and social experiences further shape and amplify the impact of this phenomenon on individuals [3]. Recognizing and addressing both these personal and environmental aspects is essential for developing effective strategies to mitigate the effects of IP.

The prevalence of IP has been established across various high-achieving professions including nursing [5, 6], psychology [1, 7], law [8], engineering [9], business [10], academia [11], and medicine [12, 13], each proposing unique approaches to tackle it. Several studies have associated IP with depression, anxiety, burnout, and perfectionism [11, 14–16]. These lead to emotional exhaustion, work-life conflict, and, in severe cases, even the risk of self-harm and suicide [17–20]. The potential impact of IP within the healthcare sector is concerning. IP is a threat to diversity [21, 22], may negatively affect patient care [23], obstruct individuals from pursuing leadership roles and further challenge their career progression [24] and professional identity within local and national organizations [25]. Hence, there is a growing need to address IP to limit its impact within healthcare.

Despite its widespread acknowledgment, there exists limited research that offers specific tactics for efficiently handling and mitigating IP. Early research suggests individual and group psychotherapy principles such as validating doubts and addressing individual fears of failure as potential methods to alleviate feelings of imposterism [1, 26, 27]. A recent systematic review found no evaluated treatment for IP [4]. However, a preliminary search indicated a recent surge of interest in addressing IP through educational interventions such as group discussions and workshops in the past five years. Therefore, this scoping review aims to provide a comprehensive summary of these interventions aimed at addressing IP across various high-achieving professional settings. The review also summarises the strategies that can help overcome IP and foster a supportive environment for healthcare professionals.

## Methods

A scoping review approach was used to achieve several objectives including examining the extent of research activity, determining the value of a full systematic review, summarising, and disseminating research findings, and identifying gaps in existing literature [28]. The review adopted the Joanna Briggs Institute (JBI) methodology [29], and used the Preferred Reporting Items for Systematic Reviews and Meta-Analyses extension for Scoping Reviews (PRISMA-ScR) guidelines [30] to report findings. The a priori protocol is registered with the Open Science Framework (OSF) [31].

### Research question

What educational interventions have been developed to address IP in high-achieving professionals, and how have they been designed and evaluated? We aimed to explore the following themes:

1. What is currently being done to address IP?
2. What is successful?
3. What is the gap for future work?

### Identifying relevant studies

An initial limited search of MEDLINE (Ovid) was performed to identify articles on the topic. The text words contained in the titles and abstracts of the relevant articles and the index terms used to describe the articles were used to develop a full search strategy (Additional file 1). The search strategy was reviewed and agreed upon by an independent librarian at the University of Sheffield. Searches were completed across MEDLINE (Ovid), SCOPUS, Web of Science, and PsycINFO databases between 10th August 2022 and 15th September 2022. Sources of unpublished studies and grey literature, including conference proceedings and research dissertations/theses were searched using Google Scholar. The reference lists of articles selected for full-text review and those finally included in the review, were also screened for additional papers. The search was not limited by a time period.

Given the scarcity of literature on interventions for IP within healthcare education research, the review extended its scope to include interventions developed in non-healthcare settings. Consequently, exploring educational interventions designed to address IP beyond healthcare systems becomes a logical progression and with a previously adopted strategy for addressing other inquiries in healthcare education [32].

The review excluded syndromes that mimic IP such as Capgras syndrome, along with studies solely focusing on the prevalence or recommending theoretical strategies to overcome IP without any description of interventions. Additionally, interventions addressing associated effects of IP, such as burnout and suicide, but without explicit mention of IP, were also excluded. Type and quality of article was not a basis for exclusion in this review.

### Study selection

A total of 427 papers were identified from databases, and 168 duplicates were eliminated using Covidence [33]. An independent reviewer (ZKS) screened titles and abstracts against the inclusion criteria. Potentially relevant papers were retrieved for full-text evaluation, and reasons for exclusion were documented. To minimise author bias and error, ZKS randomly distributed 20% of the studies at the abstract and full text review stages to be independently screened by authors HRC, RJ, and TB. A high agreement rate of 96% was achieved, and any disagreements among the reviewers were resolved through team discussion.

**Fig. 1:**
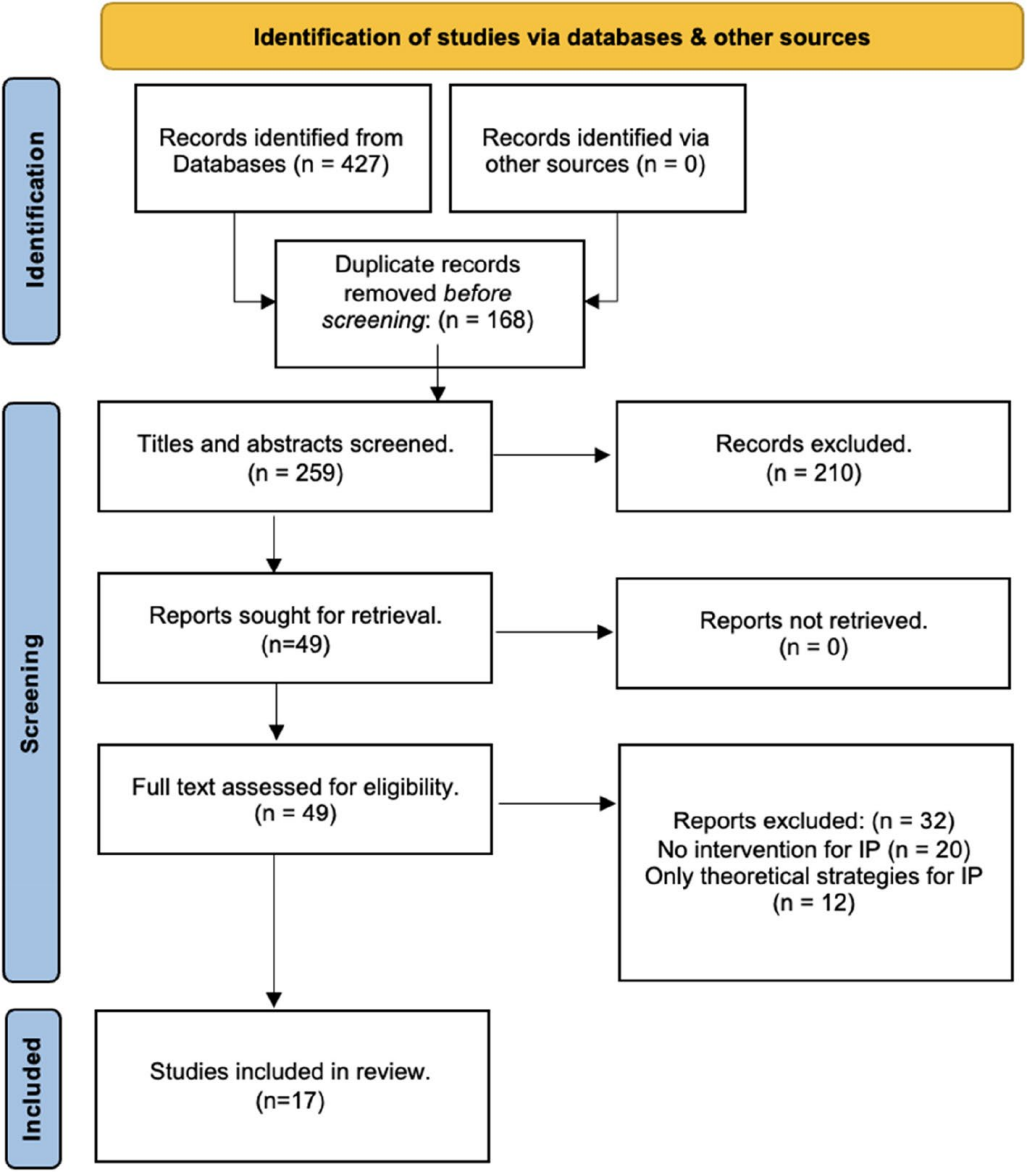
PRISMA flow chart demonstrating outcomes of search process

The PRISMA flow diagram illustrates the study selection process (Fig. 1). No additional articles were found in the grey literature search; 49 articles underwent full-text review, and 17 manuscripts met the inclusion criteria for the study.

### Collating, summarising, and reporting results

Data was extracted from included papers using a custom standardised form, on Covidence 2.0. The extracted data was analysed using MS Excel for quantitative data and NVivo for qualitative data. The finalised compiled raw data is available in Additional file 2. Collaborative analysis (ZKS and HRC) allowed comprehensive exploration into the structure and content of educational workshops (Additional file 3).

Strategies suggested and those used for addressing Imposter Phenomenon were systematically compiled from each of the included papers. The collated strategies were subsequently imported into NVIVO 12 for a more intricate thematic analysis. Following Braun & Clarke's six-step thematic analysis approach, ZKS amalgamated related codes to give rise to prospective themes (Table 2). The generated themes were subjected to scrutiny by HRC and further reviewed by the rest of the team, resulting in a unanimous agreement on their formulation.

## Results

The study characteristics are summarised in Table 1. The review revealed a notable temporal trend, with the majority of the articles published in the last three years since 2020. Majority of the studies were original research papers originating in the USA. Of the 17 papers, only 10 studies centred exclusively on healthcare. Among these, 5 studies encompassed diverse medical disciplines such as internal medicine, clinical radiology, primary care providers, psychiatry, surgery, obstetrics, and gynaecology. Two articles included undergraduate medical and dental cohorts; among the postgraduate student studies, three out of the four were related to nursing and family therapy. The professional setting in the remaining was technology, business, and STEM/academia. Two reports featured a varied professional cohort spanning the aforementioned fields as well as law and teaching. Specifically, four papers exclusively focused on females, while three others had a greater female participation than males. No interventions solely involved males, and none of the studies explored gender beyond the male/female binary.

**Table 1:** Summary of characteristics studies *(n* = *17)*

Year of publication	Frequency (% of all studies)	Reference
1978	1 (5.9)	[27]
1995	1 (5.9)	[34]
2017	1 (5.9)	[35]
2018	2 (11.8)	[36, 37]
2020	4 (23.5)	[38–41]
2021	4 (23.5)	[42–45]
2022	4 (23.5)	[46–49]
**Country**		
USA	13 (76.5)	[27, 34, 37, 39–48]
UK	2 (11.8)	[35, 49]
Canada	1 (5.9)	[36]
Austria	1 (5.9)	[38]
**Type of article**		
Research papers	13 (76.5)	[35–41, 43, 44, 46–48]
Reports	3 (17.6)	[27, 34, 42]
Dissertation	1 (5.9)	[45]
**Professional field**		
Healthcare	5 (29)	[39, 42, 43, 47, 48]
Postgraduate students	4 (23)	[35–37, 45]
Undergraduate healthcare	2 (11.8)	[41, 46]
Business and Management	2 (11.8)	[44, 49]
STEM & Academia	2 (11.8)	[40, 44]
Mixed high achieving industries	2 (11.8)	[27, 34]
Technology	1 (5.9)	[38]
**Gender**		
Males > females	3 (17.6)	[38, 41, 47]
Females > males	3 (17.6)	[44, 45, 48]
Only females	6 (35.3)	[27, 34, 35, 40, 42, 49]
Only males	0	
Other gender reported	0	
Gender not reported	5 (29)	[36, 37, 39, 43, 46]
**Intervention type**		
Group:		
Interactive workshop	10 (58.8)	[36, 37, 39, 40, 43–48]
Group psychotherapy	2 (11.8)	[27, 34]
Online training module	1 (5.9)	[41]
Individual:		
Coaching / Supervision	3 (17.6)	[38, 42, 49]
Reflective journaling	1 (5.9)	[35]

### What educational interventions have been developed to address IP?

A review of 17 papers revealed a classification into two main intervention types: individual and group-based approaches (Table 1).

#### Individual approach

Only 4 papers advocated for one-to-one intervention (Table 1). Studies recommending coaching were from fields of business, executive roles, and technology, while one-on-one structured supervision using therapy models like narrative therapy and Bowen’s Family Therapy (BFST) were utilised in training programs for healthcare trainees delivering therapy to others. An additional qualitative reflective study advocated for the effects of reflective journaling [35].

#### Group based approach

Among the 13 papers that employed a group-based approach, 10 studies were workshops, forming the majority of all identified educational interventions. Two reports proposed group psychotherapy, inspired by Gestalt psychology, outlining an intervention aimed at augmenting clients' awareness of their thoughts, emotions, and behaviours. One study assessed the effectiveness of an independent online training module designed to raise awareness of IP and provide coping strategies to address IP in dental students [41]. The studies exhibited variation in the total number of participants. Group interventions conducted multiple times encompassed a range of 65 to 396 total participants, while non-repeated interventions had a comparatively smaller sample size.

A sub-analysis was conducted to gain a deeper understanding of the educational program of the workshops, including their context and learning activities (Additional file 3).

Workshops aimed at addressing IP were conducted in diverse settings, with participant numbers ranging from small groups of 5 to larger gatherings of 100. Four papers did not specify the number of participants per workshop; however, among these, three papers provided an aggregate count of participants across multiple workshops. Some workshops were designed for multi-professional participation, while others specifically targeted individual professionals.

The duration of the group sessions ranged from approximately 30 to 120 min. Most workshops were designed to last around 60 to 90 min; however, the longest workshop occurred annually over 5 years. The duration of these workshops was adapted to accommodate different learning objectives and participant engagement strategies.

All the studies aimed to empower participants by equipping them with strategies, tools, and skills to combat IP and its negative effects. The educational objectives across the ten papers were grouped under prevalence exploration (PU), understanding and awareness of IP (U/A), intervention development (ID) and evaluation (E). All workshops use a variety of creative approaches to address IP, combining didactic presentations, interactive activities, personal reflection, and group discussions to empower participants and equip them with strategies. The learning activity for each workshop is also highlighted in Additional file 3.

### What strategies were employed and recommended?

The strategies employed in educational programs and those suggested by authors in the 17 studies were analysed thematically and categorised into individual, peer-to-peer, and institutional levels (Table 2).

**Table 2:** Thematic analysis results of strategies used to address IP in identified studies

Theme	Code	Major outcome	References
**Individual strategies**	Record Keeping	Journaling and reflecting (positive feedback, achievements, imposter thoughts)	[27, 35, 41, 46]
		Recognising strengths	[37, 39]
	Cognitive reframing	Developing growth mindset, positive thinking, and engagement, challenging negative thinking patterns, story telling	[48, 37, 38, 44, 49]
		Self-awareness, mindfulness, self-compassion	[48, 39, 45, 35, 44, 43, 49]
		Improvisation techniques, visualising success, rewarding self-accomplishments, embracing confidence	[27, 47–49]
	Support communities	Building a community for safe spaces for expression, group therapy, experiential workshops	[34, 36, 44]
		Seeking peer and mentor / supervisory support, building small networks	[37, 39, 35, 41, 43, 40, 47]
	Seeking structured supervision	Coaching for personal growth managing fear of failure	[41, 38, 49, 40]
		Exploring family of origin patterns in supervision	[27, 34, 45]
	Building personal skills	Strategic communication practices, time management, procrastination prevention, recognising stuck points	[39–41, 44]
**Peer strategies**	Acknowledgement	Understanding of IP in others with emphasis on empathy, community, and connection; allowing vulnerability	[36, 40, 41, 43]
	Breaking the silence	Challenging perfectionism and implicit bias; Celebrating peers' success, cultivating positive culture	[43, 47, 48]
**Institutional Strategies**	Creating awareness	Normalising IP feelings, exploring negative/ positive effects of IP	[44, 45, 49]
		Acknowledging the role of IP in institutional processes	[40, 45]
		Using CIPS – as a tool to guide conversation	[37, 49]
	Education on IP	Training supervisors, collaborative supervision, mentorship	[35, 42, 45, 47]
		Organisational support for coaching	[38, 49]
		Delivery of workshops, provide terminology and tools in existing curriculum	[36, 37, 39, 40, 43–48]
		Addressing systemic biases, gender equality, diversity & inclusion	[40, 47]

### How have these interventions been evaluated?

A brief overview of interventions targeting IP and the methodologies used for evaluation can be found in Table 3. Only eleven studies assessed whether the intervention had a positive effect on IP. Eight studies exclusively measured post-intervention outcomes, and three of them did not explicitly define the time frame for the evaluation after the intervention. Notably, three studies included multiple evaluation points during their described intervention [38, 41, 44]. Among these, two studies focused on measuring the distal impact of the intervention, with one assessing impact at 5 weeks using a questionnaire [38] and the other at 3 months using a questionnaire and focus group [44]. The third study assessed the impact at the "end of the semester" by administering the Clance Imposter Scale (CIPS) to measure any changes in imposter-related thoughts throughout the semester along with evaluating how students applied the six coping strategies introduced during the intervention through self-reported outcomes [41].Eight of the eleven interventional studies evaluated, used self-reported data measurements, using validated and some self-developed questionnaires using Likert scales to measure outcomes such as imposter scores, career management, goal attainment, core self-evaluation, self-efficacy, satisfaction, and perceived knowledge acquisition. Only three studies measured the distal impact of the intervention [38, 44, 45]. Furthermore, only four used qualitative methodology to evaluate the intervention (Table 3).

**Table 3:** Evaluation approaches and methodologies in the reviewed studies

**Evaluation of intervention**	**Frequency (% of all studies)**
Post-intervention only	
- Immediately	4 (24)
- 3 weeks post intervention	1(5)
- Duration not clearly defined	3 (18)
Multiple points of evaluation	3(18)
No evaluation	6 (35)
**Measure of evaluation**	**References**
Self-reported outcomes (Likert scale survey ± open response questions)	[36, 38, 39, 41, 43, 44, 47, 48]
Standardised Measurement Tools	
CIPS survey	
- Pre intervention only	[37, 40]
- Pre and post intervention	[38, 41, 44]
YIS survey	
- Pre intervention only	[43, 48]
Evaluation of outcome measures other than imposter scores	
- Goal attainment, career management, self-efficacy, tendency to cover up errors, fears of negative evaluation	[38]
- Burnout (single question)	[47]
- Core self-evaluation	[44]
- Knowledge based assessment	[38, 48]
Qualitative feedback	
- Focus groups	[44]
- Written reflections	[46]
- Semi-structured interviews	[45, 49]

Although the studies did not undergo a qualitative assessment of methodology of all the studies there was only one randomised control trial which demonstrated the effectiveness of coaching over group training in consistently reducing IP scores and fear of negative evaluation in technology trainees, along with sustained effectiveness after 5 weeks post-coaching [38]. Additionally, a qualitative study concluded a positive impact of coaching in business executives, however highlighted that the positive effects of coaching decline post-intervention [49].

## Discussion

This scoping review outlines 17 published studies concerning educational interventions aimed at addressing IP within high-achieving professions, further providing insights into strategies that can be adopted by healthcare professionals.

### What is currently being done to address IP?

The proposition of employing a combination of various therapeutic approaches, both individual and group-based, to address IP in high-achieving women, was put forth by Clance, the pioneer behind the concept of IP in 1978 [27]. Langford and Clance discuss how self-psychological theory interprets the imposter phenomenon as arising from a desire to boost self-esteem by attempting to match an idealised self-image. This behaviour is viewed as a way to cope with underlying feelings of insecurity and self-doubt. The study recommends therapeutic strategies that incorporate principles from self-psychology and cognitive therapy to address and mitigate the imposter phenomenon [7].

A review of the current literature indicates workshops are a popular means of addressing IP in high-achieving individuals particularly since 2020 (Table 1), possibly driven by the increasing focus on physician well-being [25]. Group workshops offer a platform for enhancing awareness, acknowledgment, and maintaining consistent validation [27]. Through group engagement, individuals come to recognize the prevalence of imposter feelings which alleviate symptoms of self-doubt and isolation, further are empowering individuals to challenge cognitive thinking and negative patterns [26]. Coaching and structured one-to-one supervision are other alternatives suggested by limited literature, both honing a cognitive reframing approach towards addressing IP. While coaching interventions were prominent in non-healthcare settings, healthcare professionals have specific challenges and considerations that may necessitate customising or adapting such interventions to make them suitable and effective within the healthcare context. Could such an individualistic approach place the burden of tackling a phenomenon felt by many in the hands of a few? Moreover, could it further perpetuate feelings of isolation? Individual psychotherapy on its own may imply that IP is a medical dysfunction viewing those experiencing it as patients, whereas IP is not even indexed in DSM-5 or ICD-10. Given the evidence that IP is a universal phenomenon prevalent across high-achieving professions, [1, 5–13, 15] groupwork places responsibility not only on the individual but also challenges peers and creates awareness on an institutional level. This is in line with the notion that IP is rooted not only within the family of origin but also within social context [34]. Group sessions and coaching for employees can be a strategic investment for institutions like the NHS, promoting mental health, well-being, and a positive workplace culture.

### Strategies to tackle IP

While formal recognition of IP as a medical diagnosis may enhance interventions, caution is warranted due to the potential stigma attached. Integrating IP interventions into broader practitioner health initiatives is tempting for a supportive environment, yet a tailored approach is crucial. Cumulatively, the 17 studies advocate for a collaborative approach to tackle IP using individual, peer and institutional strategies. Individual strategies can be applied within group or coaching settings, focusing on sub-themes of cognitive reframing, record keeping, building interpersonal skills, seeking structured supervision and a community for support (Table 2). The role of gender in IP remains inconclusive due to mixed evidence. While some studies suggest a potential link between higher IP scores and female gender, [41, 48] other studies [47] including a systematic review, [3] found no significant gender-related correlation with IP. Despite evidence indicating that IP can affect both men and women, the majority of interventions primarily or exclusively enrolled female participants. This pattern may not solely be attributed to gender differences but rather suggests the influence of unconscious bias and institutional challenges that women encounter in the workplace, which may perpetuate the phenomenon in women [22]. Therefore, some workshops [40, 47] advocate for a systemic cultural change. A small proportion of the selected studies call for institutional awareness of IP on a wider scale further advocating for an educational platform delivering workshops to address IP, systemic bias, and diversity and inclusion (Table 2). Additionally, four papers underscore the pivotal role of supervisors in addressing IP [35, 42, 45, 47]. Supervisors and training programme directors within the NHS play a multifaceted role in guiding, mentoring, and assessing trainees to ensure their development into competent and compassionate healthcare professionals. Their support and mentorship are vital for the success of trainees and the delivery of high-quality patient care. Therefore, educating supervisors about IP is essential for the well-being and success of trainees in healthcare training programs. Institutions should empower supervisors to recognize and address IP through collaborative supervisory relationships to support trainees going through this experience. This will ultimately contribute to the development of confident and competent healthcare professionals.

The feasibility of delivering interventions for IP within healthcare presents challenges stemming from limited resource allocation, including qualified facilitators, materials, dedicated time, and space. Nonetheless, securing support and endorsement from governing bodies, such as Health Education England, can empower program directors to establish clear policies and secure funding, thereby reinforcing the credibility and legitimacy of these interventions.

### What is successful?

Determining whether a group setting is more effective than an individual approach poses a challenging question. All 17 interventions reported positive outcomes, whether they entailed changes in CIPS scores, increased awareness and confidence in recognizing IP, or an intent to utilise discussed strategies. However, the evaluation measures employed were primarily subjective, relying on self-reported Likert scales accompanied by open-text questions. This reliance on self-reported Likert scales raises concerns about the validity of the assessment method in capturing the full scope of outcomes. Further majority of studies assessing the intervention did so immediately after its implementation. This approach captures only short-term impacts and may inadvertently introduce test–retest bias. Participants might be inclined to provide more favourable feedback immediately post-intervention due to social desirability [50]. While seven studies utilised validated CIPS scores for evaluation, it's important to note that the CIPS, despite relying on self-reported responses, is designed to serve as an objective tool for assessing the presence and severity of imposter syndrome in individuals. Researchers employ it in a systematic and standardised manner to gather data that can be analysed objectively, thereby providing valuable insights into the phenomenon of imposter syndrome within both research and clinical setting [51]. Not all the studies that incorporated CIPS consistently employ the survey before and after the intervention. This practice poses a challenge as it hinders the ability to establish a clear baseline and measure the intervention's true impact over time [52].

Only four studies tried to capture the long-term impact of the intervention, which aligns with Kirkpatrick's Level 3 evaluation, [53] assessing changes in behaviour resulting from the training. For example, an RCT evaluated the effects of coaching five weeks post-intervention, utilising validated questionnaires that assessed various facets, including IP scores, goal attainment, the inclination to conceal errors, the fear of negative evaluation, and career management [38]. Another study explored the use of BFST as a supervision tool in group therapy through semi-structured interviews conducted three weeks after the intervention [45]. Another study examined the effects of employing cognitive processing therapy tools to address IP through a follow-up validated questionnaire that assessed imposter tendencies and core self-evaluation outcomes, complemented by a one-hour follow-up focus group to delve into the transfer of learning [44]. Lastly, Metz et al. evaluated the strategies students had adopted and used over the course of the semester after the intervention [41].

Despite the well-established associations of imposter syndrome with burnout, depression, anxiety, leadership challenges, career advancement, self-efficacy, and performance, only four studies assessed the broader impact and outcomes beyond imposter scores achieved as a result of the intervention (Table 3). Among these, only two studies, namely Zanchetta et al. [38]  and Hutchins and Flores, [44] employed validated questionnaires to assess these secondary outcomes.

The strategies recommended in the selected papers for addressing IP are based on the work done by Clance and her research team [7, 26, 27, 34]. However, these strategies are often not evaluated systematically within specific professional settings to determine their effectiveness for participants. Some studies gather information about the strategies participants use in their daily lives but fail to consistently report these findings in their results. There is a lack of reporting regarding strategies suggested by participants during small group discussions [39]. When evaluations do occur, they often take place after a few weeks, with limited consideration for real-life applicability or behaviour changes [41]. Instead, participants are typically asked about their intentions to apply specific strategies rather than observing actual behavioural changes [36, 43].

### What is the gap for future work?

#### Strengths

The scoping review covers a wide range of studies related to educational interventions for addressing imposter phenomenon in high-achieving professionals, providing a comprehensive overview of the existing literature. The review employs a well-defined methodology, including the use of JBI guidelines, [ 29] to ensure rigour and consistency in the data collection and analysis process. The inclusion of studies from various high-achieving professions and settings, not limited to healthcare, allows for a broader perspective on the strategies employed to address IP. The thematic analysis of strategies used to address IP at individual, peer, and institutional levels provides valuable insights into the various approaches to tackle this phenomenon. The review highlights practical strategies and interventions that can be employed by healthcare professionals and institutions to address IP effectively, which is particularly relevant given its impact on the healthcare sector.

#### Limitations

The review may be subject to publication bias. Studies with negative or null results may not have been published, leading to an overrepresentation of positive findings. This could potentially skew the conclusions about the effectiveness of interventions. Despite efforts to conduct a comprehensive search, it is possible that some relevant studies were overlooked. Studies published in languages other than those searched may also have been missed. The scoping review focused on interventions explicitly designed to address IP. However, interventions solely targeting burnout, depression, and related psychological issues could indirectly impact IP. Excluding such interventions might have overlooked valuable insights into addressing IP within broader mental health and well-being contexts. The included studies may have employed different methodologies and evaluation techniques. Variability in study designs, outcome measures, and data collection methods can make it challenging to draw direct comparisons and generalise findings. The review encompasses non-healthcare sectors, primarily business and technology, where individual coaching has demonstrated positive effects in addressing IP. However, the applicability of these encouraging outcomes from non-healthcare to healthcare settings presents a challenge due to the substantial disparities between the two environments. Healthcare professionals navigate distinctive challenges, including high-pressure scenarios, critical life-or-death decision-making, ethical responsibilities in patient care, and the intricacies of multidisciplinary teamwork. These unique aspects differentiate their work environment significantly from the corporate world, potentially influencing the manifestation and impact of IP in distinct ways.

#### Direction for future research

Future research should aim for gender-inclusive studies to better understand how IP manifests in individuals of all genders and whether interventions need to be tailored differently. Many of the included studies rely on self-reported Likert scales for short-term evaluation, with limited analysis of comparing interventions or evaluating change in behaviour. Therefore, future research could benefit from more objective measures, longitudinal assessments, and high-quality methodology using control groups to allow for a more robust assessment of the effectiveness of interventions. Furthermore, conducting more in-depth qualitative research to explore participants' experiences, coping strategies that are effective and those that are not, and the nuances of IP in different professional settings would provide richer insights. Future research could also investigate the impact of institutional strategies, such as diversity and inclusion initiatives, in mitigating IP and creating supportive environments for professionals. Lastly, research on sustainable interventions that maintain their effectiveness over an extended period would be valuable, especially in high-stress professions like healthcare.

While this scoping review provides valuable insights into current educational interventions that predominantly address the negative aspects of IP, such as the fear of failure, perfectionism, and undermined self-esteem, it is crucial to acknowledge that certain adaptive traits contributing to feelings of IP can, at times, lead to positive outcomes. These include a strong drive for excellence and high achievement motivation [54]. This raises crucial questions about the multifaceted nature of IP. The varying ways individuals perceive and utilize their impostor feelings highlight the need for a more nuanced and comprehensive exploration of this phenomenon, aiming to inform the development of well-rounded strategies.

## Conclusion

This scoping review has summarised and synthesised existing literature on educational interventions designed to address Imposter Phenomenon in high-achieving professionals, with a particular focus on healthcare. The review examined a diverse array of interventions, including individual and group-based approaches, across various professional settings. Further recommending a hybrid approach to address IP, such as incorporating small group discussions and individual exercises as part of the intervention.

While the positive outcomes reported in the reviewed interventions are promising, the study revealed limitations in evaluation methods, often relying on self-reported data and assessing only short-term impacts. Healthcare professionals face unique challenges related to IP within their sub-speciality, necessitating the adaptation of interventions developed in other industries. Future work in this area should prioritise the development and rigorous evaluation of interventions tailored specifically to the respective professional setting. There is a need for comprehensive, long-term assessments of the interventions' effectiveness, considering their impact on behavioural changes. Systemic changes within institutions, such as promoting diversity and inclusion, educating supervisors, and establishing support networks, may play a crucial role in addressing IP effectively among high-achieving professionals in healthcare and other industries.

The review underscores the potential for a combination of individual and group-based interventions utilising individual, peer, and institutional strategies to create a supportive environment and promote the well-being and success of professionals experiencing IP.

### Supplementary Information

**Additional file 1.** Phase 1 Search in Ovid MEDLINE (conducted on August 10, 2022) and number of articles yielded.

**Additional file 2.** Raw Data Set from the Scoping Review.

**Additional file 3.** Sub analysis of workshops (n = 10) developed to address IP. Abbreviations: Prevalence exploration (PE), Understanding & awareness (U/A), Intervention development (ID), Evaluation (E), Young’s Imposter Scale (YIS), Clance Imposter Phenomenon Scale (CIPS).

## Data Availability

All data generated or analysed during this study are included in this published article and its supplementary information files.

## Abbreviations

BFST: Bowen family systems theory
CIPS: Clance imposter phenomenon scale
CSE: Core self-evaluation
E: Evaluation
IP: Imposter phenomenon
ID: Intervention development
JBI: Joanna Briggs Institute
OSF: Open science framework
PRISMA-ScR: Preferred reporting items for systematic reviews and meta-analyses extension for scoping reviews
PU: Prevalence exploration
U/A: Understanding and awareness of IP
YIS: Young’s imposter scale
