# Supplementary material for: Educational interventions for imposter phenomenon in healthcare: a scoping review
Source: BMC Med Educ. 2024 Jan 8;24:43. doi: 10.1186/s12909-023-04984-w (PMC10775670; doi:10.1186/s12909-023-04984-w)
Supplement: Supplementary file 1 — Additional file 1. Phase 1 Search in Ovid MEDLINE (conducted on August 10, 2022) and number of articles yielded. [file 12909_2023_4984_MOESM1_ESM.docx]

**Additional file 1.** Phase 1 Search in Ovid MEDLINE (conducted on August 10, 2022) and number of articles yielded.

| **Search** | **Query** | **Records retrieved** |
| --- | --- | --- |
| #1 | (Imposter Syndrome or Imposter Phenomenon or Imposterism or Impost*).mp. | 332 |
| #2 | (Interven* or Workshop or Treat* or coach* or strategy).mp | 8704290 |
| #3 | #1 AND #2 | 57 |
| - No date or language limits applied | |  |
