# Supplementary material for: Educational interventions for imposter phenomenon in healthcare: a scoping review
Source: BMC Med Educ. 2024 Jan 8;24:43. doi: 10.1186/s12909-023-04984-w (PMC10775670; doi:10.1186/s12909-023-04984-w)
Supplement: Supplementary file 2 — Additional file 2. Raw Data Set from the Scoping Review. [file 12909_2023_4984_MOESM2_ESM.docx]

**Additional file 2:** Raw Data Set from the Scoping Review.

| **Study Characteristics** | | | **Participant characteristics** | | | **Intervention** | | | |
| --- | --- | --- | --- | --- | --- | --- | --- | --- | --- |
| Reference (publication year) | Country | Type of article | Target setting | Gender  **M**ale; **F**emale | Sample size | Intervention type | Evaluation | Conclusion | Strategies |
| Clance and Imes (1978) | USA | Report | Graduate students, medical students, law, anthropology, nursing, counselling, religious education, social work, occupational therapy, and teaching | F | 150 | Individual and group psychotherapy | No evaluation | Combining multiple therapeutic approaches appears to be the most effective strategy for changing imposter beliefs in clients. Through a combination of such interventions and a commitment to change, high-achieving women can transition from feeling like imposters to embracing their accomplishments. | Developing positive behaviours towards negative feelings for IP through exercises such as record keeping of achievements  Practice Gestalt techniques such as  acting out ambitious roles  Seeking support from people who they trust and those who are not judgemental |
| Clance et al. (1995) | USA | Report | x | F | x | Group psychotherapy | No evaluation | The social and interpersonal context of a therapeutic group can offer corrective experiences that counter early messages from family and society. A group setting can be highly effective for raising awareness, exploring, and changing imposter-related feelings and behaviours, in an environment characterized by consistent validation, respectful challenges, and acceptance despite mistakes. | Understand and recognise how IP affects various personal facets of life.  Become an expert in identifying IP feelings in yourself and others.  Support each other to overcome challenges via group therapy. |
| Aird (2017) | UK | Original Research | Graduate student (nursing) | F | 1 | Reflective journaling | No evaluation | Higher education institutions should promote a learning culture that acknowledges impostership, enabling smooth transitions and facilitating support for postgraduate students. | 7 steps for **individuals**: 1. awareness of achievements + value of transferable skills 2. Speak to peers and seniors to air feelings of inadequacy. You will find you are not alone. 3. Establish communities that foster shared learning 4. Explore services that are offered to support learning before you start your programme  5. Make regular contact with your personal academic tutor or trustworthy mentor 6. Reflect on the learning and the journey, not on the outcomes 7. Practise 'writing therapy' - Journaling, reflections, poetry allows you to organise your thoughts and affirms your practice, embedding your accomplishments in reality  **Institutionally:** 1. Create infrastructure to support IP mechanisms in higher education institutions 2. Personal academic tutors should be aware and be able to recognise and support IP in students. |
| Carlisle (2018) | USA | Original Research | Graduate students (Master of Library and Information Science) | x | x | Workshop | Qualitative feedback immediately post-intervention | Workshop well received; participants relieved to find commonality in imposter feelings.  Many expressed intent to use provided tools to manage imposter syndrome in the future.  Feedback led to suggestions for workshop enhancement, including handouts and expansion to other programs. | **Suggested by participants:**  Recording positive feedback  Joining groups or volunteering  Following relevant 8-related social media accounts  Keeping the IS dialogue open with their peers |
| Haney et al. (2018) | USA | Original Research | Graduate students: (nurse practitioner and clinical nurse specialist, 4th year medical students, physical therapy, clinical counselling, speech and language, pathology,  Pharmacy,  social work, athletic training students) | x | 396 | Workshop | No evaluation | Findings only focus on IP in clinical nurse specialists.  Institutions offering clinical nurse specialist programs can easily integrate imposter syndrome content into their existing curriculum.  The use of the CIPS provides a quantifiable way to assess behaviours and characteristics that might otherwise be challenging for students to identify and label. | Strategies from literature:  Give it a name.  Talk to mentors.  Remember what you do well.  Realise that no one is perfect.  Change your thinking.  Keep learning.  Just do it |
| O'Connell et al. (2020) | USA | Original Research | STEM | F | ~100 | Workshop | No evaluation of workshop | Workshop highlights how IP influences communication and impedes career advancement.  Participants identified that their IP influenced their communication, lack of confidence, career advancement barriers, self-expression hindrance, and fearing gender-based stereotypes.  Improvisation exercises revealed how participants downplayed their accomplishments and struggled to highlight their work's importance.  Many participants seemed unaware or slightly aware of systemic bias against them, exhibiting the "not me" attitude. | **Institutional Change:** Diversity and inclusion plans with actionable strategies and measurable outcomes  Mentorship, leadership opportunities, skills-based training, and bystander training are essential to support women and minorities.  **Change in STEM:**  Building a community and peer mentoring -support networks  **Individual Approaches:**  Although women should not have to change, they should be aware of inner imposter tendencies and external biases.  Women can make empowered communication choices while staying authentic and recognizing challenges they face. |
| Zanchetta (2020) | Austria | Original Research (RCT) | Trainees in sensor technology | 70 M  33 F | 103 | Coaching vs Training intervention vs no intervention (against the background of mindset theory) | **Multiple points of evaluation**  -Pre-intervention questionnaire (included demographic questions containing gender, age, nationality, education, and work experience as well as a scale for assessing IP scores and goal attainment)  -Immediately after intervention (questions about satisfaction and utility, career management, and the mediator’s attributional style, self-efficacy, tendency to cover up errors, and fear of negative evaluation. Furthermore, we again addressed IP scores and goal attainment at this measurement point and included a MCQ questionnaire to assess content-related knowledge.  -5 weeks post-intervention (assessing IP scores, goal attainment, tendency to cover up errors, fear of negative evaluation, and career management) | Both coaching and training interventions resulted in immediate significant reductions in IP scores, with coaching demonstrating sustained effectiveness as evidenced by lower scores after 5 weeks, attributed to reduced fear of negative evaluation.  Coaching also improved self-enhancing attributions and self-efficacy, while training excelled in knowledge acquisition compared to coaching.  Coaching intervention participants showed significantly higher career management immediately as well as 5 weeks after the intervention. | Dyadic coaching sessions are particularly effective, enhancing self-enhancing attributions and self-efficacy.  Career counsellors should aid young employees in adopting a growth mindset and managing fear of failure.  Learning from failure is emphasised for personal development.  Organisations can use external/internal counsellors for individual coaching in employee development initiatives. |
| Metz et al. (2020) | USA | Original Research | 1^st^ year dental students | 55 M  48 F | 103 | Online training:  14- minute educational video with supplemental reminder cards  of 6 proposed coping mechanisms | **Multiple points of evaluation**  -Immediate evaluation (Pre-semester): Demographic and CIPS Survey following intervention.  -End of semester: Repeat of CIPS to determine any changes in imposter thoughts over the course of the semester + survey containing 6 Likert-scale responses that evaluated student use of the 6 coping mechanisms. Four open-response items asked students to discuss the impact of imposter thoughts, elaborate on the specific coping mechanisms they utilized, evaluate the tools (video, reminder cards) currently in use, and provide suggestions for revisions to the existing resources. | Intense imposter experiences decreased from 13.6% to 4.9% by semester's end.  More students exhibited few imposter characteristics, (5.8% pre-semester to 10.7% over the semester)  No impact was observed from race or age factors.  Gender differences indicated higher CIPS scores in females.  Post-semester survey: 87 / 103 students utilized activity scheduling to combat procrastination.  80/103 reduced excessive preparation time for nonessential tasks, 59 /103 engaged in discussions with peers about imposter thoughts.  56 / 103 turned their focus towards community work.  10 students sought help from mental health services.  7 students kept a notebook to document their imposter thoughts. | Six Suggested strategies:  1.Having conversations with your classmates about Imposter Thoughts  2.Keeping a notebook of your Imposter Thoughts  3.Reducing the time, you spent over-preparing for non-essential tasks  4.Scheduling your activities to prevent procrastination  5.Focusing on others through volunteer or community work  6. Seeking professional help through mental health services |
| Baumann et al. (2020) | USA | Original Research | internal medicine residents | x | x | Workshop | Immediately post-intervention: A 5-point Likert scale to assess effectiveness of intervention to promote resident wellness and three open ended questions on feeling comfortable recognising symptoms of IS in themselves and colleagues. | Of 21 respondents: 96% felt comfortable recognizing IS in themselves, 81% felt comfortable discussing IS with colleagues, and 52% felt comfortable recognizing IS in colleagues.  Additionally, 62% knew appropriate next steps after identifying IS in themselves or colleagues.  An IS wellness session was considered effective by 81%, and 76% found the facilitator instrumental in fostering discussion. However, only 47% believed they would gain more from a wellness session during an outpatient block.  Qualitatively, respondents appreciated the open discussion format around IS during the transition into new roles, valuing the presence of residents from all levels. | Seeking advice and feedback from mentors  Seeking counselling through the employee assistance program  Discussing with peers  Avoiding over preparing and procrastination  Practising self-compassion |
| Vitoria (2021) | USA | Report | Therapist trainee | F | 1 | Supervision using techniques of narrative therapy | No evaluation | Using narrative therapy improved trainee’s self-esteem, reduced shame, and fear, and helped her create a new, more positive self-narrative.  Collaboration among psychotherapy, supervision, and neuroscience fields can offer potential strategies to enhance clinicians' emotional well-being in relation to IP. | Supervision within a non-hierarchical relationship driven by mutual emotional engagement. |
| Rivera et al. (2021) | USA | Original Research | Mixed medical faculty (medical students, residents, fellows, faculty, staff, and program leadership) | x | 92 | Workshop | In session YIS survey  Immediately post-intervention: A 5-point Likert scale and four open-ended questions to evaluate the effectiveness of the workshop, including participant satisfaction, and intended behaviour change. | 92% felt the workshop met its learning objectives.  90% felt the workshop was a valuable use of their time.  89% of participants felt the supplemental handouts were useful.  90% would apply information learned at the workshop in the future.  Participants were asked to indicate two things that they would do because of the workshop. Strategies emerged on individual, peer, and institutional level.   - practicing self-compassion (21%), - seeking mentorship (15%) - embracing vulnerability (15%). - 29% aimed to enhance institutional education on IS. - 23% aimed to share their own Imposter Syndrome experiences, while 22% intended to celebrate peers' successes.   **Recommendations** for improvement: To integrate the workshop into orientations, retreats, and diversity conferences across healthcare professions and institutions.  Among the workshop components, participants preferred small-group discussions, preferably randomised, with generalised scenarios.  **Identified barriers to intended behaviours:** ingrained thought processes (11%) and self-doubt (16%) at the individual level, lack of support (22%) and openness (11%) at the peer level, and challenges changing departmental culture and workload (22%) at the institutional level. | Participants suggested:  **Individual level**  -Seek mentorship Self-compassion  -Physical reminder of accomplishments and strategies  -Allow for vulnerability  **Peer level**  -Celebrate the successes of peers  -Help others recognise IP in themselves  **Institutional level**  -Education on IS |
| Hutchins and Flores (2021) | USA | Original Research | Academic faculty: assistant professors (30%), associate professors (35%), full professors (4%), and librarians (31%).  Executives from a biotechnology firm: managers (80%) and senior executives (20%) | 42 M  58 F | 65 | Workshop | Multiple points of evaluation  -Pre workshop survey (CIPS) and Core Self-Evaluation (CSE) outcomes.  -Immediate: post workshop survey to collect participant satisfaction and intention to transfer skills (proximal)  -3 months from intervention: a second post workshop survey (imposter tendencies and self-evaluation outcomes) and 1 hour follow-up focus groups to explore distal transfer experiences and impacts. | **Change in imposter and CSE scores:** significantly lower scores post-workshop. CIPS and CSE scores were negatively correlated.  **Transfer of learning**  78% participants expressed a strong intention to use the workshop skills to address future imposter episodes.  Skills used (distal transfer):   - Recognizing the imposter mind - Challenging thinking errors to disrupt the imposter narrative. - Engaging social support as a protective factor   **Perceived impact** of using skills from workshop:   - Normalising the universality of the imposter - Increased agency over thoughts and emotions - Experiencing and witnessing change | **Suggested tools of** Cognitive Processing Therapy  Initial step involves identifying "stuck points" to counter imposter thoughts, aiding in overcoming cognitive barriers by reframing imposter episode narratives.  Sharing personal imposter experiences in groups allows for validated feelings, fosters commonality and is effective in addressing imposter thoughts. |
| Popovic M (2021) | USA | Dissertation | Graduate students taking a Master’s in Marriage & Family Therapy | 2 M  10 F | 12 | Workshop | 3 weeks post-intervention  One on one semi- structured interviews (1.5 – 2 hours) to explore impact of intervention on participant’s IP | Bowen Family Systems Theory (BFST) based group supervision positively impacted IP as students reported following impact **(themes)**  - Increased sense of awareness  - Lowered anxiety  - Increased sense of connectedness  - Increase sense of freedom  - Acquired new knowledge  Counselling supervision is key for ongoing professional development of students in therapy training who feel like imposters. | BFST emphasises collaborative relationships in therapy, supervisors should adopt a collaborative approach with their students. Foster an environment where students feel comfortable discussing their feelings of imposterism openly.  Equip students with the vocabulary and understanding to define their symptoms and experiences related to IP.  Acknowledge that IP is a common experience and not a sign of incompetence.  Structured interventions delivered timely appropriately; starting with psychoeducation to define IP and then progressing to group activities and family of origin exploration can gradually break down defensive mechanisms and increase students' sense of connectedness.   Recognize and address students' defensive mechanisms that stem from fear of being exposed as incompetent. |
| Stephens (2022) | USA | Original Research | Medical students | x | 5 | Workshop involving mask making | Duration between intervention and evaluation unknown  Thematic analysis undertaken of medical students’ reflection on comparing first mask with last mask in final year of study. | **Themes:**  **IP -** Creating masks provided students an opportunity to recognize and directly address their feelings of imposterism, acknowledging its prevalence and pervasiveness.  **Uncertainty -** concerning the evolving roles as medical practitioners and in the mask-making process itself. A shared concern was the lack of artistic confidence and worries about others' perceptions of their mask and their identity within the medical field.  **Identity Progression-** both an individual (micro) level and within broader systems (macro). As their medical school journey advanced, participants grew more comfortable in their roles as medical professionals. They also recognized that this process would likely reset as they transitioned to new roles as medical residents. | Promotes use of expression and art (mask making) as a reflection strategy to mitigate imposterism in medicine and promote healthy longitudinal professional identity formation. |
| Deshmukh et al. (2022) | USA | Original Research | Clinical radiology | 13 M  8 F | 30 | Workshop using medical improvisation techniques. | Duration between intervention and evaluation unknown  Pre-workshop online survey to assess IP prevalence, contributing factors and burnout.  Post workshop: Likert scale questionnaire to assess participant rating of workshop | **Prevalence:** 83% acknowledged experiencing IP at some point in their careers. 71% displayed frequent or intense IP symptoms.  IP not related to gender or radiology experience, but a significant correlation (p = 0.024) between IP and burnout was identified.  **Contributing factors to IP:**  Insufficient mentorship (40%)  Insufficient knowledge base (32%)  Insufficient experience (32%)  Implicit bias/discrimination/ inequity (24%)  **Post-workshop survey** showed a positive response, with an average rating of 4.4 on a scale out of 5 | Use of improvisation techniques such as paired partner activities regarding the “yes, and...” principle, and individual values affirmation exercises.  Mentorship  Increasing IP awareness |
| Ogunyemi et al. (2022) | USA | Original Research | Mixed medical faculty - 19% resident physicians, 13% physician faculty 10% medical students, and 41% academic administrators and 17% program directors  (66% of medical faculty were primary care providers, rest were from internal medicine, psychiatry, surgery, obstetrics, and gynaecology) | 40 M  138 F | 198 | Workshop | Pre-workshop: baseline knowledge test consisting of twelve items on IS + Young Imposter Syndrome (YIS) survey.  Immediately post-intervention: A perception, knowledge assessment of IS and a behaviour-based survey | 54% of medical faculty, 63% of administrators were positive for IP.  Following subtypes of IP classified: Expert 42%; Soloist 34%; Super-person 31%; Perfectionist 25%; and Natural Genius 21%. Of these three (expert, super person, perfectionist) were significantly correlated with positive IS scores.  Self-identified contributors of IP: Parent expectations 72%, female gender 58%, and academic rat race 37%.  Administrators had higher scores in career changes as a contributory risk factor, while physicians and medical students scored higher in transitions as a risk factor.  Knowledge survey scores increased from 4.94(SD=2.8) to 5.78(2.48) post-intervention(p=0.045) | Break the silence.  Separate feelings from fact - just because you feel something doesn't mean you are.  Recognise when you should feel fraudulent - recognise what is a normal response to a situation.  Accentuate the positive Develop a new response to failure and making mistakes.  Right the rules - you work under principles of 'I should always know the answer' - appreciate that you have every right as the next person to make mistakes.  Develop a new script Visualise success Reward yourself Fake it till you make it |
| Magro (2022) | UK | Original Research (Qualitative) | Business executives (CEO, managers, Directors) | F | 6 | Coaching | Duration between intervention and evaluation unknown  Semi-structured interviews to explore impact of coaching | **State before coaching:** All participants concealed their genuine emotions of inadequacy and feared exposure, putting effort into projecting confidence. Participants encountered the concept of IP leading them to seek coaching, despite lacking familiarity with coaching and having no specific anticipations.  **Coaching engagement:** Participants formed strong and positive bonds with their coaches, considering the coach-participant relationship crucial to their experiences, further finding the real value in the personal one-to-one time.  Participants felt safe with their coaches and opened more than they had anticipated.  **Impact of coaching:** All participants learned personalised practical tools that had a positive impact on IP. Participants also acknowledged: Positive long-lasting effects of coaching however, mentioned that feelings resurfaced and needed further management. Desire to share their story and coaching insights with others. | **Strategies used by participants:**  Kindness and self-talk  Storytelling and self-worth  Managing anxiety  Challenging negative thoughts  Reflecting on achievements and values Dyadic conversations and building trustworthy relationship with coach  **Other:**  Need for coaches to educate themselves on IP.  Coaches to refer clients for therapy or other forms of support if they do not feel equipped to deal with the client’s intense imposter feelings.  Normalising imposter feelings  Consider the use of CIP scale - as a method to guide conversation and to provide additional knowledge around IP; especially for those expressing resistance to the label or seeking confirmation of it from the coach. |
