# Supplementary material for: Educational interventions for imposter phenomenon in healthcare: a scoping review
Source: BMC Med Educ. 2024 Jan 8;24:43. doi: 10.1186/s12909-023-04984-w (PMC10775670; doi:10.1186/s12909-023-04984-w)
Supplement: Supplementary file 3 — Additional file 3. Sub analysis of workshops (n = 10) developed to address IP. Abbreviations: Prevalence exploration (PE), Understanding & awareness (U/A), Intervention development (ID), Evaluation (E), Young’s Imposter Scale (YIS), Clance Imposter Phenomenon Scale (CIPS). [file 12909_2023_4984_MOESM3_ESM.docx]

**Additional file 3:** Sub analysis of workshops (n = 10) developed to address IP. Abbreviations: Prevalence exploration (PE), Understanding & awareness (U/A), Intervention development (ID), Evaluation (E), Young’s Imposter Scale (YIS), Clance Imposter Phenomenon Scale (CIPS).

|  | **Context of Workshop** | | | | | | | | **Learning**  **activity** |
| --- | --- | --- | --- | --- | --- | --- | --- | --- | --- |
| **Reference (Year of publication)** | Target population | Participants per workshop | Duration of workshop | Educational objectives | | | | |  |
|  |  |  |  | PE | U/A | ID | E | Other |  |
| Carlisle (2018) | Graduate students | Not mentioned | 60  minutes | X | ✓ | ✓ | ✓ |  | Facilitators shared personal anecdotes about IP.  Participant self-reflection and group discussion on IP experiences + acquired coping skills, unique qualities, and identifying contributions to success.  Recommended future reflection on the above exercise to remind participants of their strengths and skills. |
| Stephens (2022) | Medical students | 5 | Annual workshop for 5 years (time for each workshop not mentioned) | ✓ | X | ✓ | X |  | Mask making workshop (end of each academic year for 5 years)  Reflective activity involving guided prompts comparing final masks to previous ones.  Reflections on professional identity changes, features expressed in masks, and impact of mask-making process. |
| O'Connell et al. (2020) | females in STEM (Multi professional) | 100 | 60 minutes | ✓ | ✓ | ✓ |  | Understand bias toward women in STEM | Pre-workshop assessment of IP using CIPS.  Introduction to IP  Introduction of participants: Interactive activity "The Many Whos I Am."  (Participants stood in pairs, listing things they were for one minute, then say what a trusted person would say about them)  Exploration of inner imposter influence on communication.   - Discussion of CIPS survey results. - Imposter Cover Letter Review: Analysed cover letters to highlight best practices.   Implicit bias – review of research  Institutional interventions - review of examples  4. Communication exercises: re-framing power dynamics by understanding body language and practise for an interview in a game called Half-life |
| Popovic  (2021) | Graduate students | 12 | 90 -120 minutes | X | X | ✓  * (Uses theory) | ✓ |  | Self-created IP survey to identify participants for group intervention.  Group intervention: using Bowen's Family System Theory* focusing on differentiation of self by discussing family values, connections, educational accomplishments, and coping skills.  Completion of genogram to depict multigenerational patterns (homework)  Post-workshop (3 weeks) semi-structured interviews to assess impact. |
| Hutchins and Flores (2021) | Academic faculty and executives from a biotechnology firm | 12-15 | 120 minutes | X | X | ✓  * (Uses theory) | ✓ |  | 2×120 min workshops delivered a week apart.  Pre-workshop: assessment of IP using CIPS + instructional webinar on writing imposter impact statements.  Uses 3 Cognitive processing therapy* tools:   1. A-B-C worksheet to help participants break down their impact statement. 2. The Challenging Questions worksheet: a list of questions to challenge their problematic belief. 3. The Problematic Thinking Patterns worksheet with seven patterns of thinking that reinforce negative, maladaptive beliefs, and self-defeating behaviours.   Session 1:   - Introduction to IP and CPT - Individual + group work using 3 CPT tools to work on and share impact statements, stuck points, and discussing their patterns of problematic thinking.   In between sessions, participants were asked to complete parts of the worksheets using skills presented during the first session.  Session 2: Discussion of practice assignments; continued use of CPT tools (Challenging Question, Thinking Patterns, Rewriting Impact Statement to reframe their thoughts and beliefs about the imposter episode) and sharing with a dyadic partner.  Post session survey + focus group to assess impact. |
| Haney et al. (2018) | Multi-professional  healthcare students | Not mentioned | 1 day | ✓ | ✓ | ✓ | X |  | Pre-workshop assessment of IP using CIPS.  Introduction to IP concept.  Small group discussion around IP and personal scores  Self-reflection on self-doubt, impact of IP in past, present, and future success.  Identification of strategies to reduce IS tendencies.  Use developed insight of IP to facilitate inter-professional teamwork. |
| Baumann et. al (2020) | Internal medicine residents | Not mentioned | 30 - 45 minutes | X | ✓ | ✓ | ✓ |  | Introduction to IP by reviewing data on IP in medicine.  Group discussion on why physicians are at risk of IP.  Peer-to-peer reflection on when participants felt IS symptoms (think-pair-share model)  Discussion around IP outside medicine.  Exercise to combat IP feelings, individual reflection on prior success and building strategies.  Handout featuring take-home points.  Post-workshop survey |
| Rivera et al. (2021) | Multi-professional healthcare students, doctors, faculty members, department leadership. | 6-8 | 75 minutes | ✓ | ✓ | ✓ | ✓ |  | Assessment of IP using YIS  Reflection using an interactive word cloud.  Didactic introduction on IS and optional TED Talk video.  Small and large group discussions using scenarios, based on personal experiences of the authors and their colleagues; facilitating reflection on situations that resonate with participants.  Strategies development to overcome IS on individual, peer, and institution levels.  Post-workshop survey |
| Deshmukh et al. (2022) | Radiologist | 30 | 60 minutes | ✓ | ✓ | ✓ | ✓ | To assess correlation between IP and burnout | Assessment of IP using CIPS  Paired partner activities related to the "yes, and..." principle.  Individual values affirmation exercises  Post-workshop survey |
| Ogunyemi et al. (2022) | Multi-professional healthcare students, doctors, administrators | Not mentioned | 60 - 90 minutes | ✓ | ✓ | ✓ | ✓ |  | Assessment of IP using YIS + baseline knowledge and imposter subtypes survey  Introduction to IP  Small group discussion uses scenarios  Whole group discussion on corrective strategies  Short perception, knowledge, and behaviour-based survey provided at the end. |
